# Supplementary material for: Mechanical forces couple bone matrix mineralization with inhibition of angiogenesis to limit adolescent bone growth
Source: Nat Commun. 2022 Jun 1;13:3059. doi: 10.1038/s41467-022-30618-8 (PMC9160028; doi:10.1038/s41467-022-30618-8)
Supplement: Supplementary file 2 — Supplementary Information [file 41467_2022_30618_MOESM2_ESM.pdf]

Supplementary information:

"Mechanical forces couple bone matrix mineralization with inhibition of angiogenesis to limit adolescent bone growth"

Maria Dzamukova*, Tobias M. Brunner, Jadwiga Miotla-Zarebska, Frederik Heinrich, Laura Brylka, Mir-Farzin Mashreghi, Anjali Kusumbe, Ralf Kühn, Thorsten Schinke, Tonia L. Vincent, Max Löhning*

*Corresponding authors: Max Löhning (max.loehning@charite.de),
Maria Dzamukova (maria.dzamukova@drfz.de)

Supplementary Figures

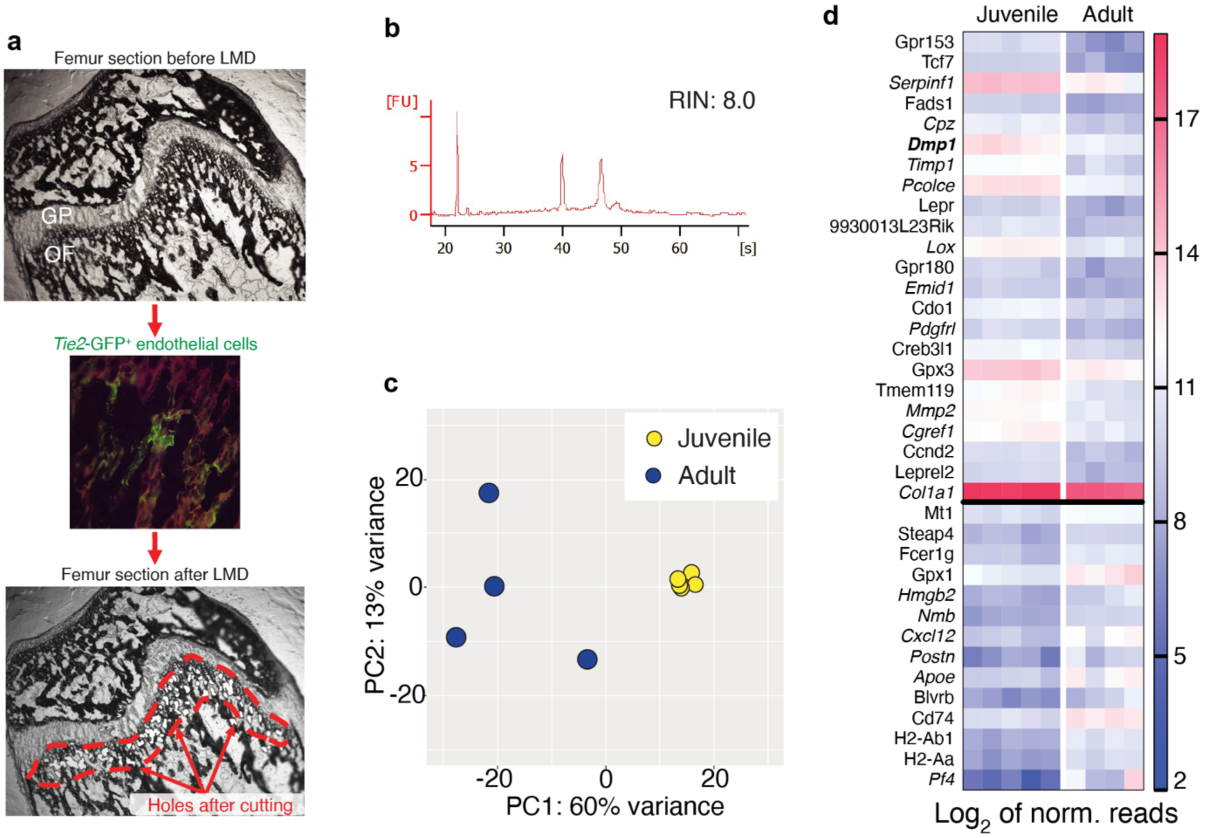

Supplementary Figure 1: Laser microdissection and sequencing of endothelial cells with surrounding cells from ossification front of juvenile and adult femurs. a, Image of the metaphysis in a section from a snap-frozen femur directly before laser microdissection (LMD) (top). Epifluorescent image of Tie2-GFP+ endothelial cells in the OF of the snap-frozen femur (middle). GFP+ regions were cut out and collected. Image of the metaphysis directly after LMD with visible holes after cutting out the capillary with surrounding cells, which were collected for further transcriptome analysis via next-generation sequencing (NGS) (bottom). **b**, Bioanalyzer plot showing good RNA integrity number (RIN) of RNA isolated from laser-microdissected sample from femur section processed according to the protocol we developed. The sample consisted of larger laser-microdissected pieces from OF, since the amount of RNA in the real sample (single capillary with surrounding cells) was below the detection limit of Bioanalyzer. **c**, Principal component analysis of the juvenile (4-week-old) and adult (12-week-old) capillary with surrounding cells from OF, which we obtained via LMD, showing that most of the difference is derived from the different sample groups and the heterogeneity within the group increases with ageing. **d**, Heat map of the most highly and significantly differentially regulated genes in the juvenile versus adult group, where dentin matrix protein 1 (*Dmp1*) was identified as an interesting candidate for further analysis. Genes encoding ECM proteins are set in *italics*. Mean expression value >500, Log_2 FC > 0.66, $P < 10^{-5}$.

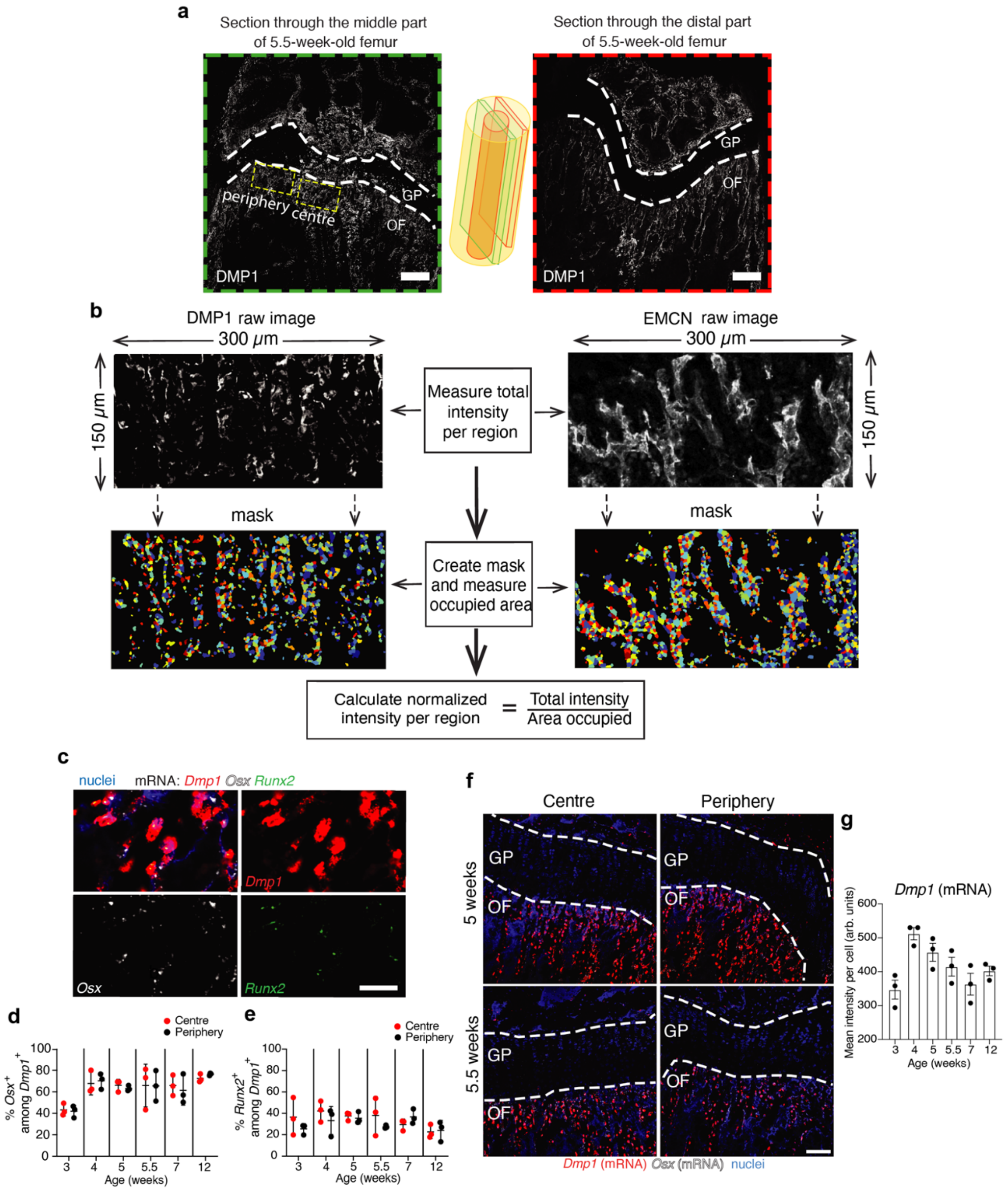

Supplementary Figure 2: Osteoblasts are the main DMP1 producer in the metaphysis and automated quantification strategy of histological images. a, DMP1 immunostaining of sections taken from the middle (left image, green plane in the scheme) or distal (right image, red plane in the scheme) part of the femur. Yellow dashed rectangles indicate the analysis areas that were defined as centre and periphery and used for further automated quantification. **b**, Scheme explaining the automated quantification strategy of images using CellProfiler software. First, the total intensity of the raw image, which is always the same size ($4.5x10^4$ μm^2), was measured and then the area occupied by the protein was calculated by creating a mask. Then the normalized intensity was calculated by dividing the total intensity by the occupied area (DMP1, EMCN) or by the total cell density (FAM20C). When the total amount of a protein per region was analysed, we used the total intensity without further normalization to the area occupied by it (DMP1, pVEGFR2, MMP9). Background subtraction using imageJ software was always done before quantification. **c**, *Dmp1*, *Osx* and *Runx2* RNAScope staining in the OF of a 5-week-old femur. Scale bar 20 μm. **d**,**e**, Quantification of (**d**) *Osx*$^+$ and (**e**) *Runx2*$^+$ cells among *Dmp1*-expressing cells based on RNAScope images. n=3 per age. Pooled data from 2 independent experiments. Mean ± SD. **f**, *Dmp1* and *Osx* RNAScope overview images from central and peripheral parts of the metaphysis at 5 and 5.5 weeks demonstrating the absence of *Dmp1* expression in the GP. **g**, Quantification of the mean *Dmp1* intensity per cell among *Dmp1*-expressing cells showing the peak at 4 weeks. n=3 per age, $4x10^5$ μm^2 area was always analysed per sample. Pooled data from 2 independent experiments. Mean ± SEM. **d,e,g,** Source data are provided in Source Data file.

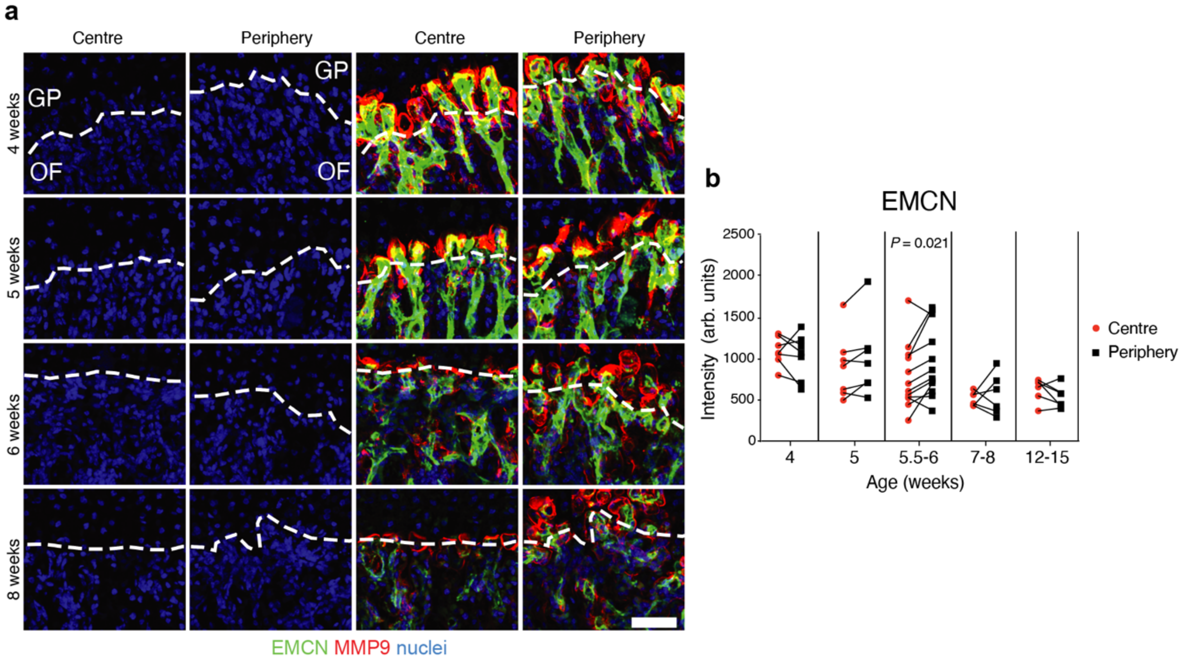

Supplementary Figure 3: Identification of bulges and loss of type H vessels starting from 5.5-6 weeks of age in the centre of the ossification front. a, EMCN and MMP9 immunostaining with a separated nuclei channel (Hoechst, left panel), which was used to define the border (dashed line) between OF and GP, based on cell density. This border then was copied to the respective full-colour image (right panel) to identify the bulges in the GP in an unbiased way. Scale bar 50 μm. **b**, Quantification of EMCN intensity in the centre and periphery of the OF at different ages with a depiction of individual values per mouse in pairs. EMCN brightness decreases with ageing. At 4 and 5 weeks n=7, 5.5-6 weeks n=12, 8 weeks n=6, 12-15 weeks n=6. Pooled data from 6 independent experiments. Two-tailed paired *t*-test. Source data are provided in Source Data file.

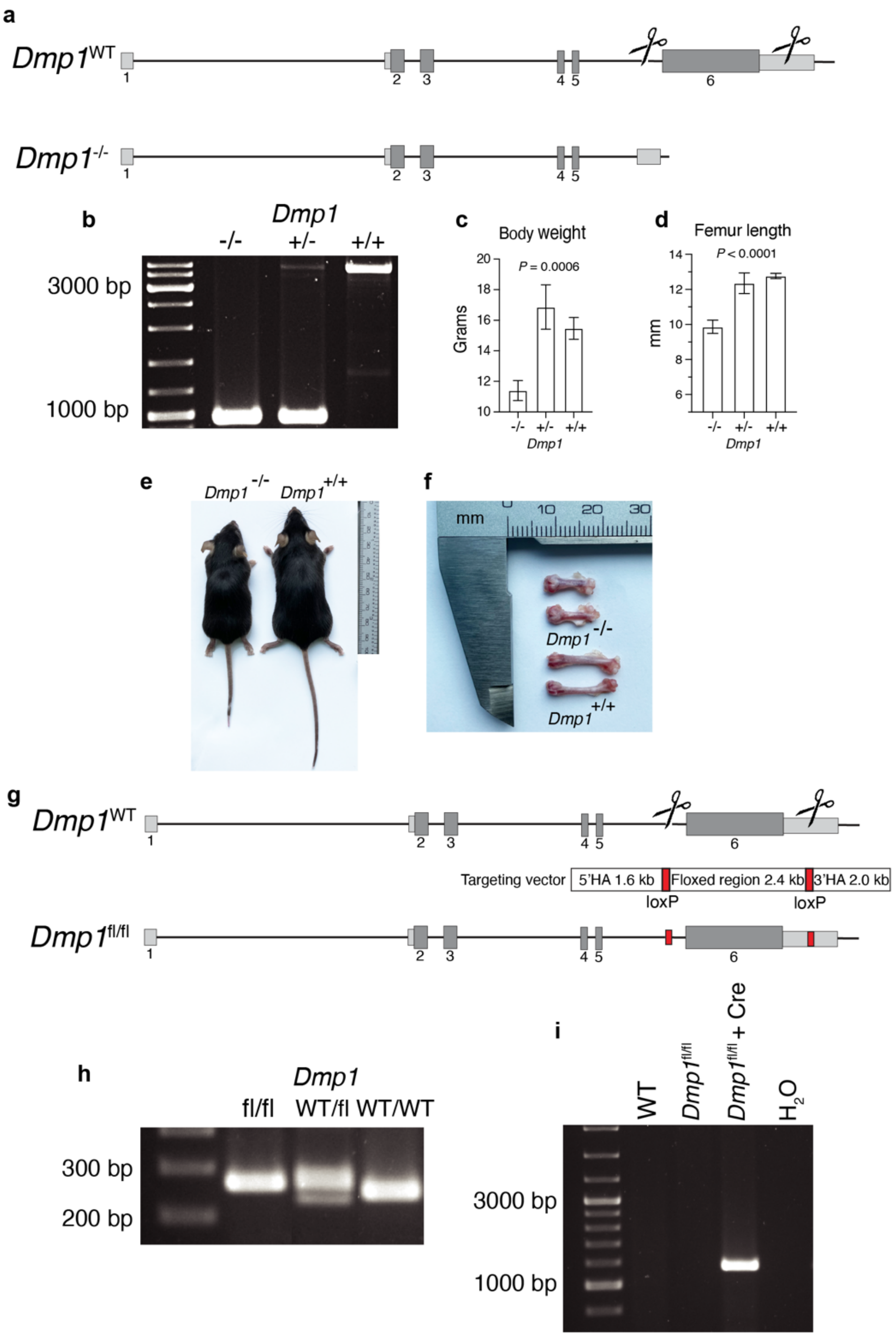

Supplementary Figure 4: Generation of *Dmp1*-deficient and *Dmp1*-floxed mice. a, Scheme showing the wild-type (top) and knockout version (bottom) of the *Dmp1* gene. Exon 6, encoding 80% of the protein, was cut out using Crispr/Cas9 technology. The cutting was done 500bp upstream of Exon 6 and 500bp downstream of the open reading frame (ORF), and the whole intermediate region was removed. **b**, PCR bands of the *Dmp1* gene in *Dmp1*$^{-/-}$, *Dmp1*$^{+/-}$ and *Dmp1*$^{+/+}$ mice, where the band at ≈3800bp indicates the WT allele and at ≈1100bp the *Dmp1*-deficient variant. Note that the wild-type band in *Dmp1*$^{+/-}$ mice is very faint because of the PCR competition with the significantly smaller knockout allele. **c**,**d**, Body weight (**c**) and femur length (**d**) of *Dmp1*$^{-/-}$, *Dmp1*$^{+/-}$ and *Dmp1*$^{+/+}$ mice at the age of 4.5-5 weeks. *P* values indicate the significance between *Dmp1*$^{-/-}$ versus *Dmp1*$^{+/-}$ and *Dmp1*$^{+/+}$ mice. Two-tailed *t*-test. Source data are provided in Source Data file. **e**, Representative photo of *Dmp1*$^{-/-}$ and *Dmp1*$^{+/+}$ mice at the age of 15 weeks. **f**, Representative photo of *Dmp1*$^{-/-}$ and *Dmp1*$^{+/+}$ femurs at the age of 5 months. **g**, Scheme showing the wild-type (top) and floxed version of the *Dmp1* gene. LoxP sites were introduced 500bp upstream of Exon 6 and 500bp downstream of the ORF in the UTR region using Crisp/Cas9 technology. **h**, PCR bands of the *Dmp1* gene in *Dmp1*$^{fl/fl}$, *Dmp1*$^{WT/fl}$ and *Dmp1*$^{WT/WT}$ mice showing that the introduced loxP sites make the allele 34bp longer than the WT version. **i**, PCR showing the appearance of the genomic *Dmp1*-knockout band only in cells from *Dmp1*$^{fl/fl}$ mice that were transduced with a Cre-recombinase encoding retrovirus.

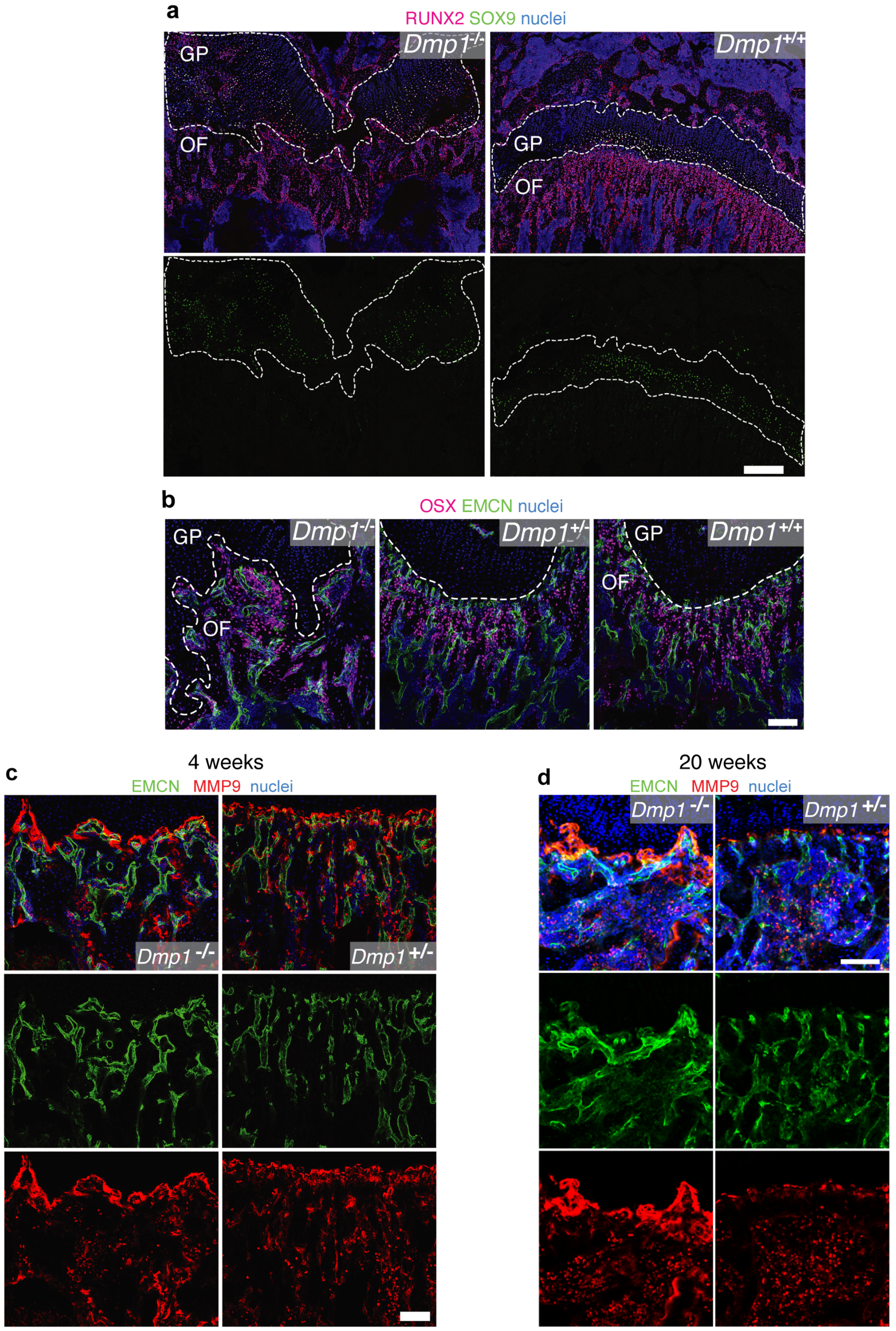

Supplementary Figure 5: DMP1-deficient mice exhibit very high EMCN and MMP9 intensity and the growth plate is expanded and disorganized with enlarged SOX9 and RUNX2 zones. **a**, RUNX2 and SOX9 immunostaining of metaphysis from 4.5-week-old *Dmp1*$^{-/-}$ and *Dmp1*$^{+/+}$ mice. Scale bar 250 μm. **b**, OSX and EMCN immunostaining of femurs from 4.5-week-old *Dmp1*$^{-/-}$, *Dmp1*$^{+/-}$ and *Dmp1*$^{+/+}$ mice from one litter. Scale bar 100 μm. **c**,**d**, EMCN and MMP9 immunostaining of 4-week-old (**c**) and 20-week-old (**d**) femurs of *Dmp1*$^{-/-}$ and *Dmp1*$^{+/-}$ mice from one litter. Scale bar 100 μm (c) and 200 μm (d).

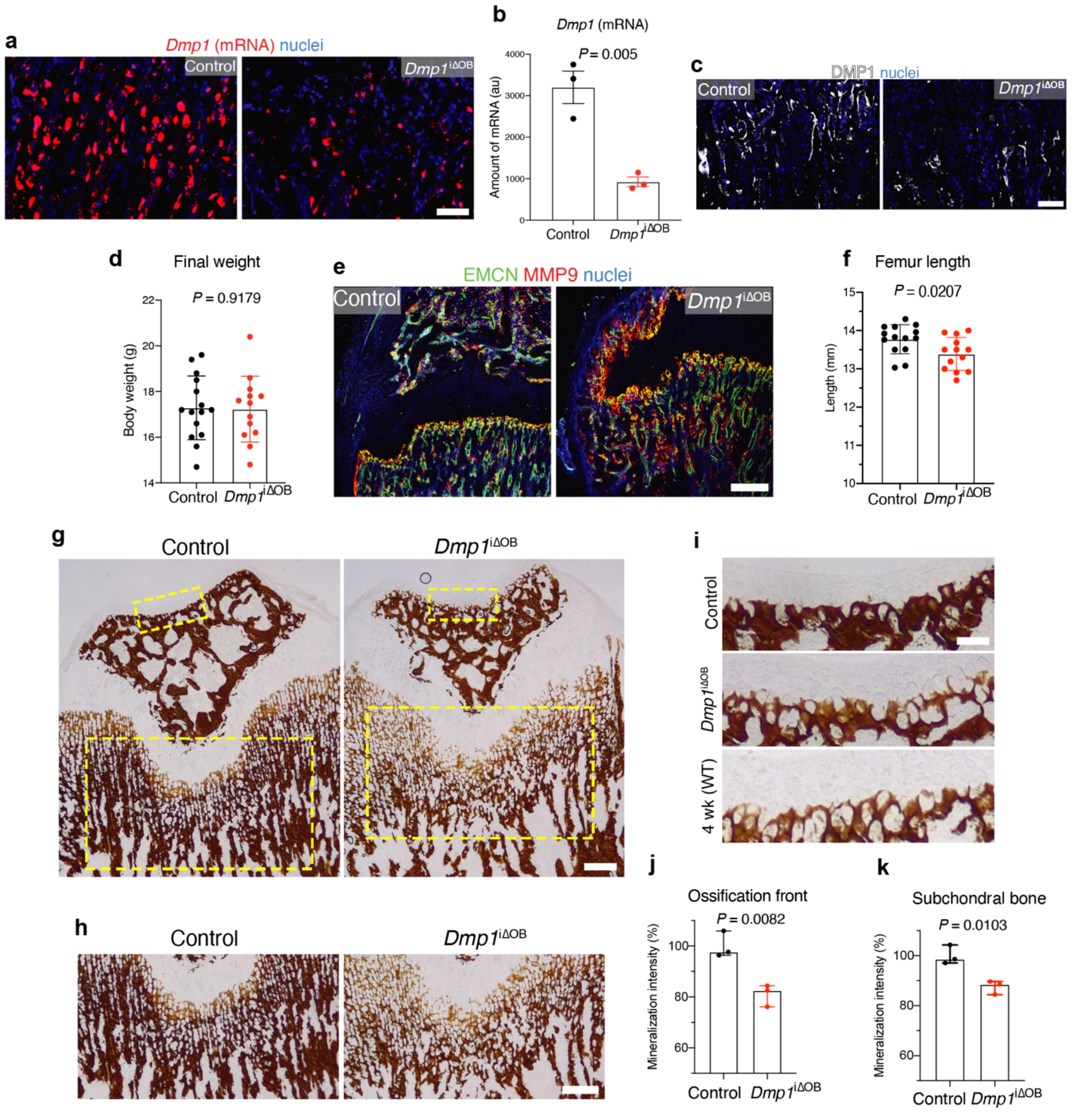

Supplementary Figure 6: Osteoblast-specific *Dmp1* deletion preserves type H vessel identity and impairs bone mineralization. a, Representative *Dmp1* RNAScope images of control and $Dmp1^{i\Delta OB}$ OF. Scale bar 50 μm. **b**, Quantification of *Dmp1* mRNA based on RNAScope images of control and $Dmp1^{i\Delta OB}$ mice at 6 weeks. 3 samples per group were randomly selected, out of 14 and 13, respectively, for control and $Dmp1^{i\Delta OB}$ groups, to determine the deletion efficiency. Two-tailed unpaired *t*-test. Mean ± SD. **c**, DMP1 immunostaining of OF in 6-week-old control and $Dmp1^{i\Delta OB}$ femurs. Scale bar 50 μm. **d**, Body weight of control and $Dmp1^{i\Delta OB}$ mice at 6 weeks (P42). **e**, EMCN and MMP9 immunostaining of 6-week-old control and $Dmp1^{i\Delta OB}$ femurs. Scale bar 250 μm. **f**, Femur length of control and $Dmp1^{i\Delta OB}$ mice at 6 weeks (P42). **d**,**f**, Pooled data from 3 independent experiments. Control n=14, $Dmp1^{i\Delta OB}$ n=13. Two-tailed unpaired *t*-test. Mean ± SD. **g**, Overview of von Kossa staining of undecalcified control and $Dmp1^{i\Delta OB}$ femur sections. Yellow dashed rectangles indicate the regions shown in Supplementary Figure 6h and 6i, and quantified in Supplementary Figure 6j and 6k, respectively. Scale bar 200 μm. **h**, Von Kossa staining of undecalcified control and $Dmp1^{i\Delta OB}$ femur sections (ossification front). Scale bar 200 μm. **i**, Von Kossa staining of undecalcified control and $Dmp1^{i\Delta OB}$ femur sections (subchondral bone – articular cartilage area). Scale bar 50 μm. **j**,**k**, Quantification of mineralization rate in the ossification front (**j**) and subchondral bone (**k**) by measuring the von Kossa staining intensity in control and $Dmp1^{i\Delta OB}$ femurs. Age = 6 weeks. Control n=3, $Dmp1^{i\Delta OB}$ n=3. Two-tailed unpaired *t*-test. Median ± range. **b,d,f,j,k,** Source data are provided in Source Data file.

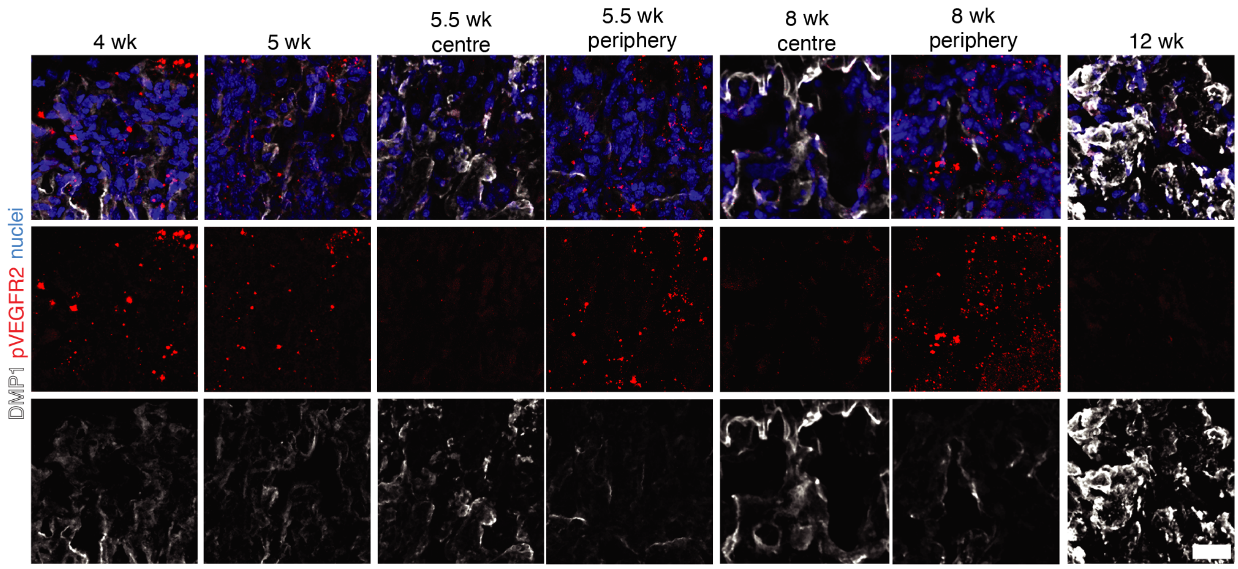

Supplementary Figure 7: DMP1 abundance negatively correlates with the activation of VEGFR2 in the ossification front. DMP1 and phosphorylated VEGFR2 (pVEGFR2) immunostaining of the OF throughout postnatal development. Scale bar 50 μm.

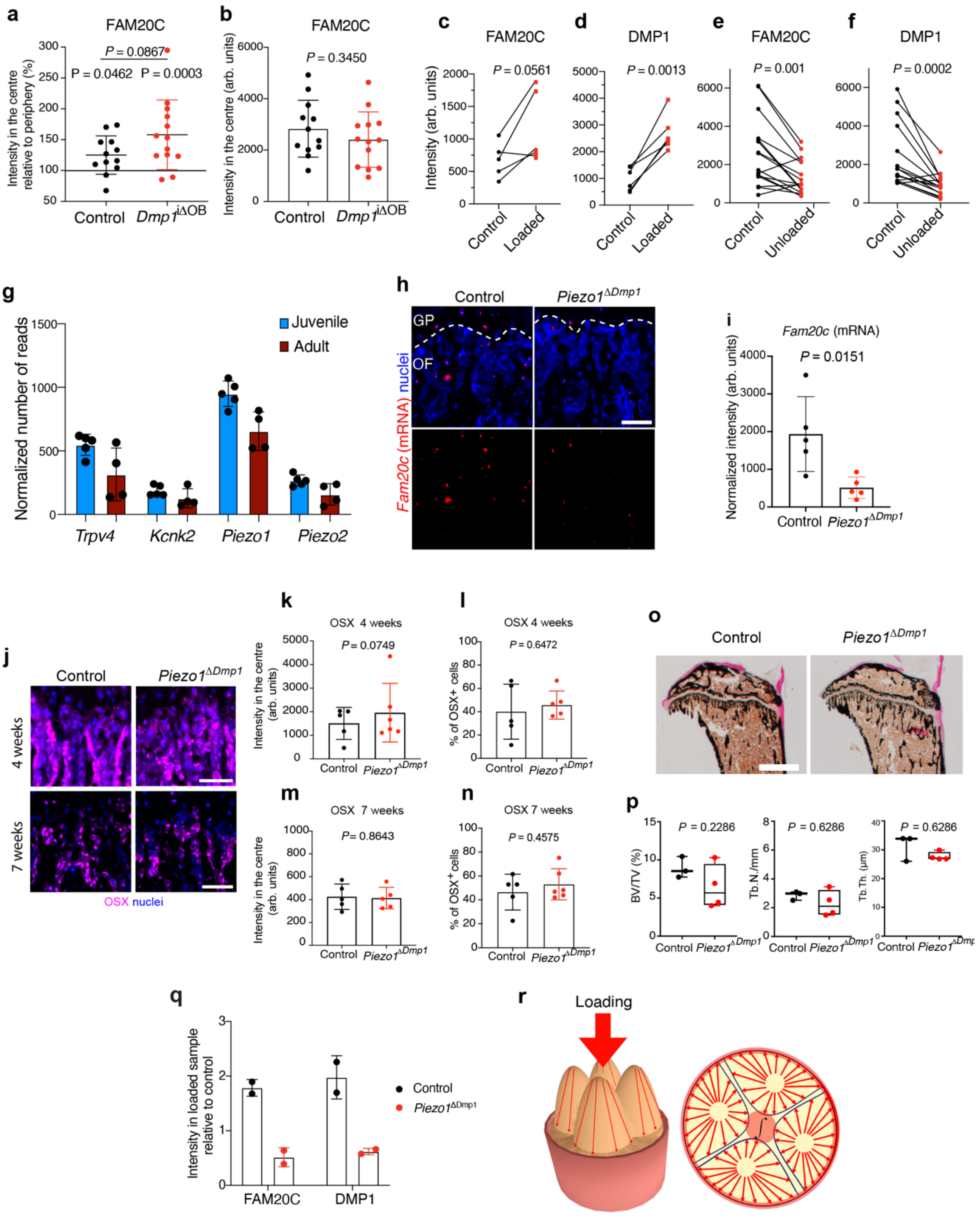

Supplementary Figure 8. Enhanced FAM20C production depends on mechanical forces sensed via PIEZO1 mechanoreceptor. a, Quantification of the FAM20C amount in the centre relative to the periphery in the OF of femurs from 6-week-old control and *Dmp1*$^{\mathrm{i}\Delta\mathrm{OB}}$ mice. Top *P* value indicates the insignificance between control and *Dmp1*$^{\mathrm{i}\Delta\mathrm{OB}}$ groups. Bottom *P* values show the significance between the intensity in the centre versus periphery upon paired analysis for every mouse. Two-tailed paired *t*-test within a group. Two-tailed unpaired *t*-test between the groups. **b**, Quantification of the total FAM20C amount in the centre of the OF in the control and *Dmp1*$^{\mathrm{i}\Delta\mathrm{OB}}$ group. Two-tailed unpaired *t*-test. **a**,**b**, Pooled data from 3 independent experiments. Control n=12, *Dmp1*$^{\mathrm{i}\Delta\mathrm{OB}}$ n=13. Mean ± SD. **c**,**d**, Quantification of the intensities of FAM20C (**c**) and DMP1 (**d**) in control and loaded (centrifuged) femur slice samples. Pooled data from 2 independent experiments. n=5-6 (in one biological replicate staining for FAM20C was technically not possible due to the limited amount of material). Two-tailed paired *t*-test. **e**,**f**, Quantification of FAM20C (**e**) and DMP1 (**f**) intensities in loaded control (left) and unloaded (operated, right) femurs after double neurectomy. Pooled data from 2 independent experiments. n=16. Two-tailed Wilcoxon matched-pairs signed rank test. **g**, Normalized number of reads mapping to the genes of mechanoreceptors expressed by endothelial cells

together with surrounding cells in the juvenile and adult OF. Data are derived from the transcriptome analysis shown in Fig. 1 and Supplementary Figure 1. **h**, Representative *Fam20c* RNAScope images of control and *Piezo1*$^{\Delta Dmp1}$ OF. Scale bar 50 μm. **i**, Quantification of *Fam20c* mRNA based on RNAScope images of 7-week-old control and *Piezo1*$^{\Delta Dmp1}$ OF. n=5. Two-tailed unpaired *t*-test. **j**, OSX immunostaining of control and *Piezo1*$^{\Delta Dmp1}$ OF at 4 and 7 weeks of age. Scale bar 50 μm. **k,m**, Quantification of OSX intensity in the OF of control and *Piezo1*$^{\Delta Dmp1}$ OF at 4 weeks (**k**) and 7 weeks (**m**) of age. n=5-6. Two-tailed unpaired *t*-test. Mean ± SD. **l**,**n**, Quantification of the frequency of OSX-positive cells in the OF of control and *Piezo1*$^{\Delta Dmp1}$ femurs aged 4 weeks (**l**) and 7 weeks (**n**). **o**, Von Kossa/van Gieson staining of 7-week-old control and *Piezo1*$^{\Delta Dmp1}$ tibias. Scale bar 1 mm. **p**, Quantification of the trabecular bone volume per tissue volume (BV/TV) (left), trabecular number per mm (Tb.N./mm) (middle) and trabecular thickness (Tb. Th. (μm)) (right) of 7-week-old control and *Piezo1*$^{\Delta Dmp1}$ tibias by histomorphometry. Control n=3, *Piezo1*$^{\Delta Dmp1}$ n=4. Two-tailed Mann Whitney test. **q**, Quantification of FAM20C and DMP1 intensities in the loaded samples relative to the control (non-centrifuged) group. n=2 per group, every dot represents an average of 2 technical replicates per biological replicate. **r**, Schematic depiction of loading distribution (red arrows) in the growth plate in 3D (left) and in projection (right). **a-g,i,k-n,p-q,** Source data are provided in Source Data file.

Supplementary tables

Supplementary Table 1. Primary antibodies used in the study.

Name	Dilution	Catalogue number	Company
Endomucin (V.7C7)	1:100	sc-65495	Santa Cruz Biotechnology
DMP-1	1:100	AF4386	R&D systems
OSX (A-13)-R	1:200	sc-22536-R	Santa Cruz Biotechnology
MMP-9, biotin	1:200	BAF909	Novus
FAM20C	1:100	25395-1-AP	Proteintech
RUNX2 [EPR14334] (Alexa Fluor® 647)	1:200	ab215955	Abcam
SOX9 [EPR14335] (Alexa Fluor® 594)	1:200	ab202517	Abcam
Phospho-VEGF Receptor 2 (Tyr1175) (19A10)	1:100	2478S	Cell Signaling
VEGFR2/KDR/Flk-1	1:50	AF644	R&D systems
VEGFR3/Flt-4	1:50	AF743	R&D systems

Supplementary Table 2. Secondary antibodies used in the study.

Name	Dilution	Catalogue number	Company
Donkey anti-Rabbit IgG (H+L) Secondary Antibody, Alexa Fluor 555	1:500	A-31572	Thermo Fisher Scientific
Goat anti-Rabbit IgG (H+L) Secondary Antibody, DyLight 633	1:400	35562	Thermo Fisher Scientific
Donkey anti-Rabbit IgG (H+L) Secondary Antibody, Alexa Fluor® 594 conjugate	1:500	A-21207	Thermo Fisher Scientific
Donkey anti-Rabbit IgG (H+L) Cross-Adsorbed Secondary Antibody, DyLight 488	1:400	SA5-10038	Thermo Fisher Scientific
Donkey Anti-Sheep IgG NorthernLights™ NL493-conjugated Antibody	1:200	NL012	R&D systems
Donkey Anti-Sheep IgG NorthernLights™ NL637-conjugated Antibody	1:200	NL011	R&D systems
Donkey anti-Sheep IgG (H+L) Secondary Antibody, Alexa Fluor® 633 conjugate	1:500	A-21100	Thermo Fisher Scientific
Donkey anti-Rat IgG (H+L) Cross Adsorbed Secondary Antibody, DyLight 488 conjugate	1:400	SA5-10026	Thermo Fisher Scientific
Donkey anti-Rat IgG (H+L) Cross Adsorbed Secondary Antibody, DyLight 550 conjugate	1:400	SA5-10027	Thermo Fisher Scientific
Donkey anti-Rat IgG (H+L) Cross-Adsorbed Secondary Antibody, DyLight 594	1:400	SA5-10028	Thermo Fisher Scientific
Donkey anti-Goat IgG (H+L) Alexa Fluor 633	1:500	A-21082	Thermo Fisher Scientific
Streptavidin AF-555	1:2000	S32355	Thermo Fisher Scientific
